# The effect of concomitant DPPIVi use on glycaemic control and hypoglycaemia with insulin glargine 300 U/mL (Gla-300) versus insulin glargine 100 U/mL (Gla-100) in people with type 2 diabetes: A patient-level meta-analysis of EDITION 2 and 3

**DOI:** 10.1371/journal.pone.0190579

**Published:** 2018-01-25

**Authors:** Jean-François Yale, Jeremy Hodson Pettus, Miguel Brito-Sanfiel, Fernando Lavalle-Gonzalez, Ana Merino-Trigo, Peter Stella, Soazig Chevalier, Raffaella Buzzetti

**Affiliations:** 1 Department of Medicine, McGill University, Montreal, Canada; 2 Department of Endocrinology, University of California, San Diego, United States of America; 3 University Hospital Puerta de Hierro, Majadahonda, Spain; 4 University Hospital, Universidad Autónoma de Nuevo León, San Nicolás de los Garza, Mexico; 5 Sanofi, Paris, France; 6 Sanofi, Chilly-Mazarin, France; 7 Department of Experimental Medicine, Sapienza University of Rome, Rome, Italy; Florida International University Herbert Wertheim College of Medicine, UNITED STATES

## Abstract

**Aims:**

To evaluate the effect of concomitant dipeptidyl peptidase IV inhibitor (DPPIVi) use on efficacy and safety of insulin glargine 300 U/mL (Gla-300) versus glargine 100 U/mL (Gla-100) in people with type 2 diabetes on oral antihyperglycaemic drugs.

**Methods:**

A post hoc patient-level meta-analysis was performed using data from EDITION 2 (basal insulin [N = 811]) and EDITION 3 (insulin-naïve [N = 878]), multicentre, randomised, open-label, parallel-group, phase 3a trials of similar design. Endpoints analysed included HbA_1c_, hypoglycaemia and adverse events, investigated in subgroups of participants with and without concomitant DPPIVi use.

**Results:**

Of 1689 participants randomised, 107 (13%, Gla-300) and 133 (16%, Gla-100) received DPPIVi therapy. The least squares mean change in HbA_1c_ (baseline to month 6) was comparable between treatment groups, irrespective of DPPIVi use (no evidence of heterogeneity of treatment effect across subgroups, p = 0.753), although group sizes were unbalanced. The cumulative mean number of confirmed (≤3.9 mmol/L [≤70 mg/dL]) or severe hypoglycaemic events, and the risk and annualised rate of such events, were consistently lower for Gla-300 than Gla-100 during the night (between 00:00 and 05:59 h) or at any time of day (24 h period), irrespective of DPPIVi use. Severe hypoglycaemia occurred in 8/838 and 10/844 participants in the Gla-300 and Gla-100 groups, respectively, and was not affected by DPPIVi use. The adverse event profile was similar between treatment groups and DPPIVi subgroups.

**Conclusions:**

Glycaemic control with Gla-300 was comparable to Gla-100, with less hypoglycaemia during the night and at any time of day (24 h), irrespective of concomitant DPPIVi use.

**Trial registration:**

ClinicalTrials.gov NCT01499095; NCT01676220

## Introduction

Type 2 diabetes is a chronic, progressive disease that requires complex management strategies [1]. People with type 2 diabetes typically start monotherapy with biguanides (e.g. metformin) [2]. However, as the disease progresses, most patients will require combination therapy with one or more oral antihyperglycaemic drugs (OADs), and/or non-insulin injectable antihyperglycaemic drugs, as well as basal insulin or more complex insulin regimens, to compensate for the diminishing insulin secretory capacity [2].

Insulin glargine 300 U/mL (Toujeo®, Sanofi, Paris, France [Gla-300]) is a basal insulin, approved in many countries, which has a more stable and prolonged pharmacokinetic profile than insulin glargine 100 U/mL (Lantus®, Sanofi, Paris, France [Gla-100]) [3]. The safety and efficacy of Gla-300 compared with Gla-100 was investigated in 1689 participants with type 2 diabetes receiving concomitant OADs in the EDITION 2 (background therapy basal insulin + OAD) and EDITION 3 (background therapy OAD only, insulin-naïve) studies [4, 5]. For those receiving sulphonylureas, use of these medications was stopped prior to randomisation and prohibited during both studies. Results from these studies have shown that Gla-300 provides comparable glycaemic control to Gla-100 with less hypoglycaemia, over 6 months of treatment [4, 5].

Other than biguanides, which were used by more than 90% of participants, the most common class of OADs in the EDITION 2 and 3 studies was dipeptidyl peptidase IV inhibitor (DPPIVi) therapy. DPPIVi therapy is becoming increasingly popular in the treatment of type 2 diabetes because it is associated with a lower risk of hypoglycaemia compared with sulphonylureas or glinides [6]. Previous studies of DPPIVi and basal insulin use in patients with type 2 diabetes have shown that this combination therapy leads to improved glycaemic control compared with basal insulin alone without an increase in hypoglycaemia, and with little or no effect on body weight or insulin dose [7–13].

This post hoc patient-level meta-analysis aimed to assess the combination of DPPIVi therapy with Gla-300, and compared the efficacy and safety of Gla-300 and Gla-100 with or without concomitant DPPIVi use in people with type 2 diabetes in the EDITION 2 and EDITION 3 studies, over 6 months of treatment.

## Participants and methods

### Study design and participants

EDITION 2 (NCT01499095) and EDITION 3 (NCT01676220) were multicentre, randomised, open-label, two-arm, parallel group, treat-to-target phase 3a studies forming part of the EDITION programme. Full study methods and results for these studies have been published previously [4, 5]. Both studies enrolled adults ≥18 years of age with type 2 diabetes. In EDITION 2, participants on prior basal insulin ≥42 U/day plus OADs with HbA_1c_ ≥53 to ≤86 mmol/mol (≥7.0 to ≤10.0%) were enrolled. In EDITION 3, participants who were on OADs (and insulin-naïve) with HbA_1c_ ≥53 to ≤97 mmol/mol (≥7.0 to ≤11.0%) were enrolled. In brief, participants were randomised (1:1) to once-daily subcutaneous injections of Gla-300 (using a modified SoloSTAR® [EDITION 2] or a modified Tactipen® [EDITION 3] pen-injector) or Gla-100 (using a SoloSTAR® pen-injector), administered at the same time each evening. Evening was defined as the period from immediately prior to the evening meal until bedtime. Gla-300 and Gla-100 were titrated to a fasting self-monitored plasma glucose target of 4.4–5.6 mmol/L (80–100 mg/dL). Appropriate local or national ethics committees approved the study protocols, and both studies were registered with ClinicalTrials.gov and conducted according to Good Clinical Practice and the Declaration of Helsinki.

Although EDITION 2 and EDITION 3 were conducted in different populations of people with type 2 diabetes, the consistent study designs (as described above) and endpoints allowed a pooled analysis to be performed. For this post hoc analysis of EDITION 2 and 3, the consistency of results was examined across four subgroups of the pooled populations (participants receiving either Gla-300 or Gla-100, with or without concomitant DPPIVi therapy).

### Outcomes

The pooled analysis of the effect of DPPIVi use was carried out for the following endpoints: change from baseline to month 6 in mean HbA_1c_ (% [NGSP units]), percentage of participants achieving HbA_1c_ <7.0%; daily basal insulin dose (U/kg) and body weight (kg); percentage of participants with ≥1 hypoglycaemic event, hypoglycaemic event rates per participant-year and cumulative mean number of hypoglycaemic events per participant (during the night [00:00–05:59 h] or at any time of day [24 h]); and adverse events during the 6-month study period. Confirmed or severe hypoglycaemia combined three American Diabetes Association definitions of hypoglycaemia [14]: documented symptomatic hypoglycaemia (typical symptoms of hypoglycaemia accompanied by a measured plasma glucose concentration of ≤3.9 mmol/L [≤70 mg/dL] or <3.0 mmol/L [<54 mg/dL]), asymptomatic hypoglycaemia (no symptoms of hypoglycaemia, but confirmed by a measured plasma glucose concentration of ≤3.9 mmol/L [≤70 mg/dL] or <3.0 mmol/L [<54 mg/dL]), and severe hypoglycaemia (an event requiring the assistance of another person to actively administer carbohydrate, glucagon or other resuscitative actions).

### Data analysis and statistics

Change in HbA_1c_ from baseline to month 6 was analysed using a mixed model for repeated measurements approach. The model included fixed categorical effects of treatment group, visit, treatment-by-visit interaction, randomisation strata of screening HbA_1c_ (<8, ≥8%), and study, as well as the continuous fixed covariates of baseline HbA_1c_ value and baseline HbA_1c_ value-by-visit interaction. Homogeneity of treatment effect across the two trials was also assessed. For hypoglycaemic events, relative risks were analysed using a Cochran–Mantel–Haenszel method, and rate ratios were analysed using an overdispersed Poisson regression model. Cumulative mean numbers of hypoglycaemic events per participant were evaluated using Nelson–Aalen estimates.

A heterogeneity test was performed to assess treatment effect across DPPIVi use subgroups and p-values were generated using a subgroup-by-treatment interaction. Differences between subgroups were considered significant if the subgroup-by-treatment interaction p-value was <0.05.

Insulin dose, baseline characteristics, percentage of participants achieving HbA_1c_ <7.0%, adverse events and body weight were analysed descriptively.

Efficacy endpoints were analysed according to the treatment group assigned at randomisation and using the modified intention-to-treat (mITT) population, defined as all randomised participants who received at least one dose of study drug and had both a baseline and at least one post-baseline assessment. Safety endpoints were analysed according to the treatment actually received and using the safety population, which included all participants randomised and exposed to at least one dose of study drug.

## Results

### Study population

Overall, 1689 participants (the majority of whom were on biguanides [1562, 92%]) were randomised to treatment in the EDITION 2 and EDITION 3 studies. Of these 736/843 (87%) in the Gla-300 group and 713/846 (84%) in the Gla-100 group did not receive concomitant DPPIVi therapy; 107 (13%) and 133 (16%) participants, respectively, were receiving DPPIVi therapy. The mITT population comprised 1670 participants (Gla-300: n = 835 [without DPPIVi: n = 728 (87%); with DPPIVi: n = 107 (13%)]; Gla-100: n = 835 [without DPPIVi: n = 702 (84%); with DPPIVi: n = 133 (16%)]).

Baseline characteristics, including concomitant OAD use, are summarised in Table 1. Baseline characteristics were similar within both the ‘without DPPIVi’ and ‘with DPPIVi’ subgroups. The majority of participants on concomitant DPPIVi therapy received sitagliptin phosphate (77% each in the Gla-300 and Gla-100 groups; Table 2).

**Table 1:** Baseline demographic and clinical characteristics, by concomitant DPPIVi use (pooled randomised population).

	Participants without concomitant DPPIVi use		Participants with concomitant DPPIVi use	
	Gla-300 (N = 736)	Gla-100 (N = 713)	Gla-300 (N = 107)	Gla-100 (N = 133)
**Age, years**	57.7 ± 9.4	57.4 ± 9.8	60.4 ± 10.2	60.4 ± 9.7
**Gender (male), n (%)**	379 (51.5)	359 (50.4)	61 (57.0)	80 (60.2)
**Racial group, n (%)**				
Caucasian Black Asian/Oriental Other	643 (87.4) 55 (7.5) 27 (3.7) 11 (1.5)	617 (86.5) 63 (8.8) 26 (3.6) 7 (1.0)	82 (76.6) 9 (8.4) 15 (14.0) 1 (0.9)	104 (78.2) 10 (7.5) 18 (13.5) 1 (0.8)
**Body weight, kg**	97.2 ± 22.9	96.4 ± 21.3	94.2 ± 22.6	98.3 ± 24.0
**BMI, kg/m**^**2**^	33.9 ± 6.8	33.9 ± 6.2	32.6 ± 6.9	34.2 ± 7.2
**Estimated GFR, mL/min/1.73 m**^**2**^	82.4 ± 20.0	81.8 ± 20.4	76.5 ± 23.8	74.2 ± 19.2
Duration of diabetes,* years	11.2 ± 6.7	11.0 ± 6.8	12.1 ± 8.1	11.2 ± 6.4
**Duration of basal insulin,**^**†**^ **years**	6.2 ± 5.4	6.4 ± 5.1	5.2 ± 4.5	5.2 ± 4.3
**Prior basal insulin dose,**^**‡**^ **U/kg/day**	0.66 ± 0.21	0.69 ± 0.26	0.71 ± 0.29	0.65 ± 0.22
**HbA**_**1c**_				
mmol/mol % (NGSP units)	69 ± 11 8.4 ± 1.0	69 ± 11 8.4 ± 1.0	66 ± 9 8.2 ± 0.9	66 ± 9 8.2 ± 0.8
**Concomitant antihyperglycaemic medication use,**^**§**^ **n (%)**				
Biguanides GLP-1 receptor agonists Sulphonylureas^¶^ Thiazolidinediones	697 (94.7) 22 (3.0) 26 (3.5) 23 (3.1)	676 (94.8) 17 (2.4) 27 (3.8) 25 (3.5)	80 (74.8) 0 (0.0) 2 (1.9) 3 (2.8)	109 (82.0) 1 (0.8) 5 (3.8) 15 (11.3)

Data are pooled from EDITION 2 and EDITION 3, and are presented as mean ± SD unless otherwise indicated

*Without DPPIVi use: Gla-300 N = 731, Gla-100 N = 711, with DPPIVi use: Gla-300 N = 107, Gla-100 N = 132

^†^EDITION 2 study only, without DPPIVi use: Gla-300 N = 372, Gla-100 N = 354, with DPPIVi use: Gla-300 N = 32, Gla-100 N = 53

^‡^EDITION 2 study only, without DPPIVi use: Gla-300 N = 350, Gla-100 N = 334, with DPPIVi use: Gla-300 N = 29, Gla-100 N = 50

^§^Participants could be on >1 antihyperglycaemic medication (EDITION 2: use of sulphonylureas prohibited within 2 months prior to screening and during study; EDITION 3: sulphonylureas, glinides and other OADs not approved for use with insulin were discontinued at baseline)

^¶^Prior use of sulphonylurea was reported in 275 (33%) of Gla-300-treated participants and 268 (32%) of Gla-100-treated participants; the majority of these participants (Gla-300: 247/275 [89.8%]; Gla-100: 236/268 [88.1%]) discontinued sulphonylurea use at randomisation as per protocol. BMI, body mass index; DPPIVi, dipeptidyl peptidase IV inhibitor; GFR, glomerular filtration rate; GLP-1, glucagon-like peptide 1; NGSP, National Glycohemoglobin Standardization Program; OAD, oral antihyperglycaemic drug

**Table 2:** Type of concomitant DPPIVi therapy used (pooled randomised population).

DPPIVi type, n (%)	Gla-300 (N = 107)	Gla-100 (N = 133)
Sitagliptin	82 (76.6)	103 (77.4)
Saxagliptin	12 (11.2)	15 (11.3)
Linagliptin	8 (7.5)	5 (3.8)
Vildagliptin	6 (5.6)	10 (7.5)

Data are pooled from EDITION 2 and EDITION 3. DPPIVi, dipeptidyl peptidase IV inhibitor

### Glycaemic response

**Fig 1:**
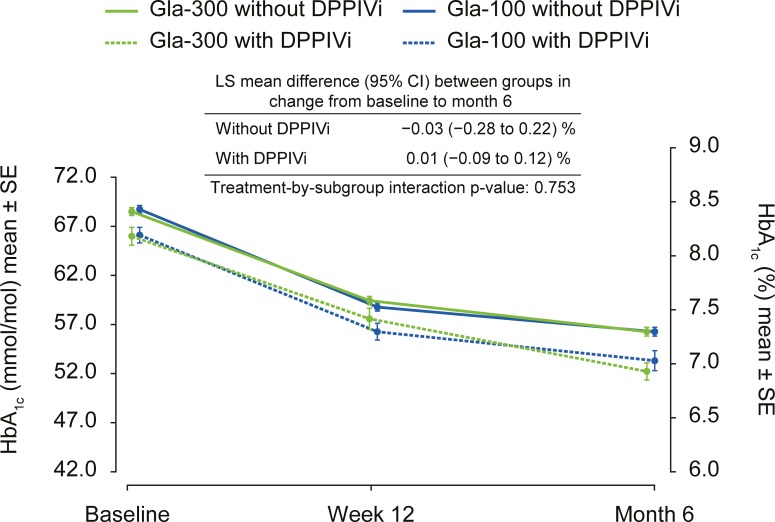
HbA_1c_ (mean ± SE) during 6 months of treatment, by visit and DPPIVi use (pooled mITT population). Data are pooled from EDITION 2 and EDITION 3. CI, confidence interval; DPPIVi, dipeptidyl peptidase IV inhibitor; LS, least squares; mITT, modified intention-to-treat; SE, standard error.

A comparable decrease in HbA_1c_ was observed in both treatment groups, irrespective of DPPIVi use (Fig 1). The mean ± standard deviation (SD) HbA_1c_ reduction between baseline and month 6 was 12 ± 12 mmol/mol (1.1 ± 1.1%) in the Gla-300 without DPPIVi subgroup, 13 ± 11 mmol/mol (1.2 ± 1.0%) in the Gla-300 with DPPIVi subgroup, 12 ± 13 mmol/mol (1.1 ± 1.2%) in the Gla-100 without DPPIVi subgroup and 12 ± 10 mmol/mol (1.1 ± 0.9%) in the Gla-100 with DPPIVi subgroup. The least squares mean change from baseline to month 6 was comparable between insulin treatment groups, in participants who did or did not receive concomitant DPPIVi therapy (no evidence of heterogeneity of treatment effect across subgroups, p = 0.753; Fig 1). The study-by-treatment interaction was found to be non-significant.

In those receiving Gla-300, the number of participants achieving HbA_1c_ <7.0% at month 6 was 267 (38.4%) and 50 (50.0%) in the without and with DPPIVi subgroups, respectively. Corresponding numbers of Gla-100-treated participants were 248 (37.2%) and 62 (47.0%), respectively.

### Insulin dose

The mean ± SD daily average basal insulin dose increased numerically in both treatment groups, to 0.78 ± 0.34 U/kg for Gla-300 without DPPIVi, 0.73 ± 0.35 U/kg for Gla-300 with DPPIVi, 0.71 ± 0.30 U/kg for Gla-100 without DPPIVi and 0.59 ± 0.29 U/kg for Gla-100 with DPPIVi, at month 6 (S1 Table). The majority of the increase took place in the first 12 weeks of treatment. In participants who did not receive DPPIVi therapy, the mean (SD) change from baseline to month 6 in daily average basal insulin dose was 0.36 ± 0.26) U/kg for Gla-300 and 0.27 ± 0.24 U/kg for Gla-100 (S1 Table). Corresponding values in participants who received DPPIVi were 0.41 ± 0.28 U/kg and 0.23 ± 0.20 U/kg (S1 Table).

### Hypoglycaemia

#### Confirmed (≤3.9 mmol/L [≤70 mg/dL]) or severe hypoglycaemia

**Fig 2:**
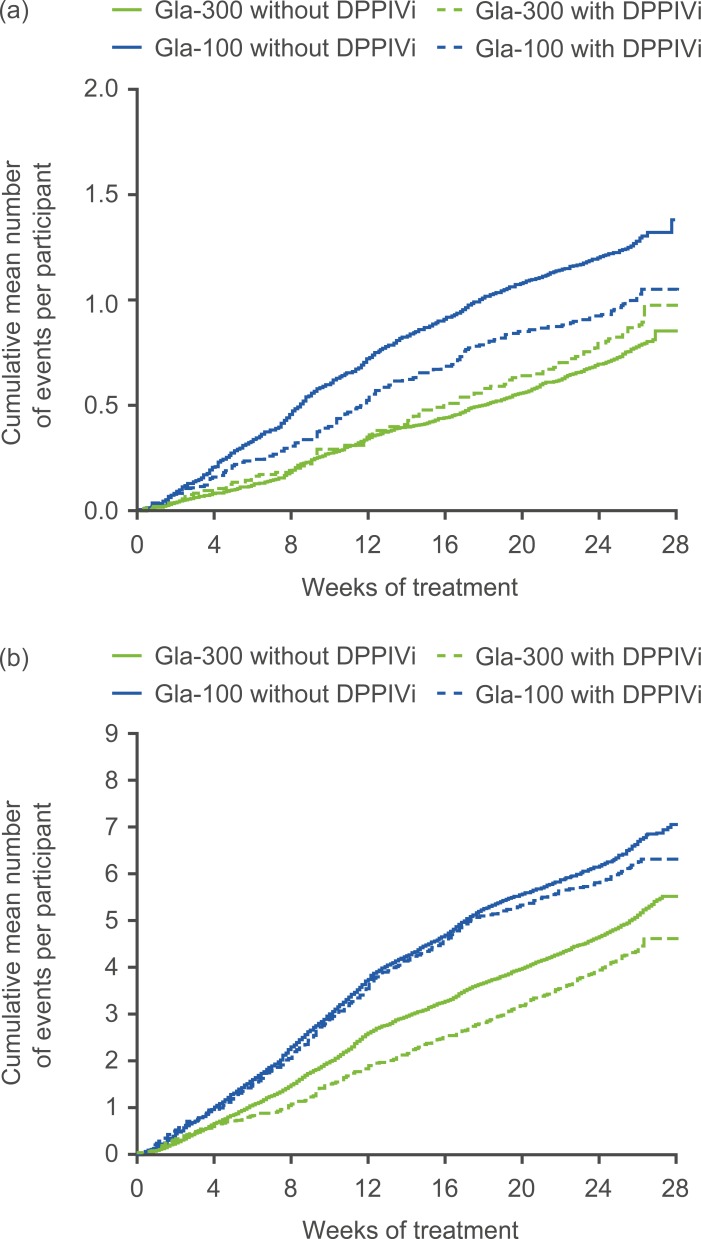
**Cumulative mean number of confirmed (≤3.9 mmol/L [≤70 mg/dL]) or severe hypoglycaemic events per participant, by DPPIVi use: (a) nocturnal (00:00–05:59 h) events; (b) any time of day (24 h) events (pooled safety population).** Data are pooled from EDITION 2 and EDITION 3. DPPIVi, dipeptidyl peptidase IV inhibitor.

The cumulative mean number of confirmed (≤3.9 mmol/L [≤70 mg/dL]) or severe hypoglycaemic events per participant remained lower with Gla-300 versus Gla-100 over 6 months, both at night (00:00–05:59 h) and at any time of day (24 h), regardless of DPPIVi use (Fig 2).

**Fig 3:**
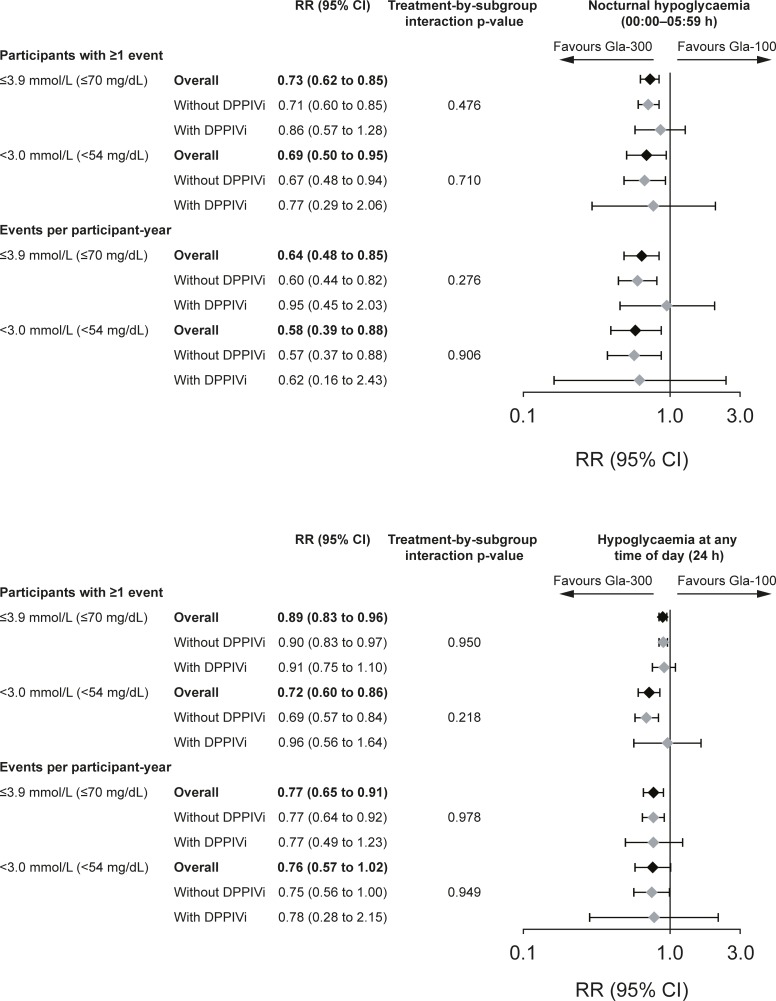
Confirmed or severe hypoglycaemia over 6 months, by DPPIVi use (pooled safety population). Data are pooled from EDITION 2 and EDITION 3. CI, confidence interval; DPPIVi, dipeptidyl peptidase IV inhibitor; RR, relative risk for participants with ≥1 event or rate ratio for events per participant-year.

The risk of ≥1 confirmed (≤3.9 mmol/L [≤70 mg/dL]) or severe hypoglycaemic event was consistently lower with Gla-300 than with Gla-100, at night (00:00–05:59 h) and at any time of day (24 h), regardless of DPPIVi use (no evidence of heterogeneity of treatment effect across subgroups, p = 0.476 [nocturnal events]; p = 0.950 [events at any time of day]) (S2 Table and Fig 3). For example, the risk of experiencing ≥1 nocturnal confirmed (≤3.9 mmol/L [≤70 mg/dL]) or severe hypoglycaemic events was reduced by 29% (relative risk 0.71 [95% CI: 0.60 to 0.85]) in the ‘without DPPIVi’ subgroup and by 14% (relative risk 0.86 [95% CI 0.57 to 1.28]) in the ‘with DPPIVi’ subgroup. In the ‘with DPPIVi’ subgroup, the reduction did not achieve statistical significance as the upper bound of the 95% CI exceeded 1. The risk of experiencing ≥1 any time of day (24 h) confirmed (≤3.9 mmol/L [≤70 mg/dL]) or severe hypoglycaemic events was reduced by approximately 10% in both the ‘without’ (relative risk 0.90 [95% CI: 0.83 to 0.97] and ‘with’ (relative risk 0.91 [95% CI: 0.75 to 1.10]) DPPIVi subgroups, although statistical significance was not achieved in the ‘with’ subgroup.

The annualised rate of confirmed (≤3.9 mmol/L [≤70 mg/dL]) or severe hypoglycaemic events at night (00:00–05:59 h) and at any time of day (24 h) was also lower with Gla-300 than with Gla-100, regardless of DPPIVi use (no evidence of heterogeneity of treatment effect across subgroups, p = 0.276 [nocturnal events]; p = 0.978 [events at any time of day]) (S2 Table and Fig 3). The rate of nocturnal confirmed (≤3.9 mmol/L [≤70 mg/dL]) or severe hypoglycaemic events was reduced by 40% in the ‘without DPPIVi’ subgroup (rate ratio 0.60 [95% CI: 0.44 to 0.82]) and by 5% in the ‘with DPPIVi’ subgroup (rate ratio 0.95 [95% CI: 0.45 to 2.03]). Any time of day (24 h) confirmed (≤3.9 mmol/L [≤70 mg/dL]) or severe hypoglycaemic events rates were reduced by 23% in both DPPIVi subgroups, although the upper bound of the 95% CI exceeded 1 in the ‘with DPPIVi’ subgroup (S2 Table and Fig 3).

A consistent pattern was observed for the incidence and annualised rate of confirmed or severe hypoglycaemic events at the stricter <3.0 mmol/L (<54 mg/dL) threshold (S2 Table and Fig 3). For both nocturnal (00:00–05:59 h) and any time of day (24 h) hypoglycaemic events there was no evidence of heterogeneity of treatment effect according to DPPIVi subgroup: p = 0.710 and p = 0.218 (incidence of nocturnal and any time of day [24 h] events, respectively) and p = 0.906 and 0.949 (rate of nocturnal and any time of day [24 h] events, respectively). As with the less stringent glucose threshold, individual reductions in risks for the stricter (<3.0 mmol/L [<54 mg/dL]) threshold achieved statistical significance in the ‘without DPPIVi’ subgroup and did not achieve significance in the ‘with DPPIV’ subgroup (S2 Table and Fig 3).

#### Severe hypoglycaemia

Severe hypoglycaemia occurred in 8/838 (1.0%) participants in the Gla-300 group (without DPPIVi: 6/731 [0.8%]; with DPPIVi: 2/107 [1.9%]) and 10/844 (1.2%) participants in the Gla-100 group (without DPPIVi: 9/711 [1.3%]; with DPPIVi: 1/133 [0.8%]). The relative risk (95% confidence interval [CI]) for Gla-300 versus Gla-100 was 0.82 (0.33 to 2.00) overall, 0.67 (0.25 to 1.80) for the ‘without DPPIVi’ subgroup and 1.40 (0.29 to 6.62) for the ‘with DPPIVi’ subgroup. The concomitant use of DPPIVi had no impact on the incidence of severe hypoglycaemia (no evidence of heterogeneity of treatment effect across subgroups, p = 0.298).

### Weight change

In participants not receiving DPPIVi therapy, mean ± SD body weight change was 0.26 ± 3.74 kg and 0.83 ± 3.39 kg in the Gla-300 and Gla-100 group, respectively. In participants receiving concomitant DPPIVi therapy, mean ± SD body weight change from baseline to month 6 was 0.68 ± 3.63 kg in the Gla-300 group and 0.37 ± 3.46 kg in the Gla-100 group.

### Adverse events

The adverse event profile was similar between subgroups, irrespective of concomitant DPPIVi use (S3 Table).

## Discussion

The results of this post hoc patient-level meta-analysis of the EDITION 2 and EDITION 3 studies were consistent with those observed in the individual study populations [4, 5]. The HbA_1c_ reduction over the 6-month study period was comparable between the Gla-300 and Gla-100 groups, irrespective of concomitant DPPIVi use. Additionally, Gla-300 was associated with a lower risk of experiencing ≥1 confirmed (≤3.9 mmol/L [≤70 mg/dL]) or severe hypoglycaemic event than Gla-100, both at night and at any time of day, regardless of DPPIVi use (no significant difference between treatments by subgroup interaction: p = 0.476 and 0.950, respectively). Overall, the results from this analysis suggest that combination therapy with Gla-300 and DPPIVi is efficacious and well tolerated.

With incretin-based therapies such as DPPIVi becoming increasingly popular in the treatment of type 2 diabetes, it is of clinical importance to assess the efficacy and safety of the combination of DPPIVi therapy with basal insulin. This topic has been addressed in a number of randomised controlled trials that have evaluated the administration of DPPIVi in previously DPPIVi-naïve patients with type 2 diabetes who were poorly controlled on basal insulin therapy [8, 10]. However, in contrast to the studies considered in this analysis, the basal insulin dose was pre-specified to remain stable in most of these studies [8], probably because these studies were designed to evaluate the effect of the DPPIVi rather than that of the insulin. Comparisons between results from these studies of DPPIVi use and those from the analysis presented here should therefore be made with caution. The current post hoc analysis adds to the limited body of evidence in this setting, and is the first study to evaluate combination therapy with DPPIVi and the recently approved Gla-300. Only one study by Mathieu *et al*. has investigated the co-administration of sitagliptin (the DPPIVi most commonly used by participants in the EDITION studies in this analysis) with Gla-100 in a treat-to-target trial in people with type 2 diabetes [9].

Owing to the treat-to-target design of the EDITION trials, HbA_1c_ change from baseline was comparable with Gla-300 and Gla-100, for both the ‘with DPPIVi’ and ‘without DPPIVi’ subgroups. This comparable reduction in HbA_1c_ (LS mean treatment difference 0.01% ‘with DPPIVi’ and −0.03% ‘without DPPIVi’), accompanied by the lower risk of hypoglycaemia with Gla-300 versus Gla-100, was associated with a numerically higher daily insulin dose for Gla-300, regardless of DPPIVi use (note statistical testing was not performed on insulin dose). Differences between the DPPIVi use subgroups were seen in Gla-100-treated participants, with the daily basal insulin dose at month 6 being numerically lower for participants on concomitant DPPIVi therapy compared with those who were not. The results observed in the Gla-100 group are aligned with those of the Mathieu *et al*. study, in which participants receiving sitagliptin required a lower dose of Gla-100 than those receiving placebo [9]. In most other studies of the co-administration of basal insulin and DPPIVi, the dose of insulin remained essentially unchanged [7, 8, 11, 12]. Nevertheless, it should be noted that differences in the numbers of people in the ‘with DPPIVi’ and ‘without DPPIVi’ subgroups (mITT population: with DPPIVi: n = 240; without DPPIVi: n = 1430) in the current analysis limit interpretation.

As indicated by non-statistically significant treatment-by-subgroup interaction p-values, the lower risk and lower annualised rate of confirmed (≤3.9 mmol/L [≤70 mg/dL]) or severe hypoglycaemia (nocturnal [00:00–05:59 h, p = 0.476 and p = 0.276; respectively] and at any time of day [24 h], p = 0.950 and p = 0.978; respectively) with Gla-300 versus Gla-100 seen in the EDITION 2 and EDITION 3 studies was observed in this analysis irrespective of concomitant DPPIVi use. The cumulative mean number of confirmed (≤3.9 mmol/L [≤70 mg/dL]) or severe hypoglycaemic events was also consistently lower with Gla-300 versus Gla-100, irrespective of concomitant DPPIVi use, both at night and at any time of day. This finding is consistent with most other studies of combination therapy with basal insulin and DPPIVi, which have reported no increased risk of hypoglycaemia in participants who received a DPPIVi plus insulin compared with those who received insulin alone [8, 10]. In the analysis presented here, the magnitude of the lower risk of hypoglycaemia for Gla-300 versus Gla-100 was generally consistent whether DDPIVi therapy was present or not, although this lower risk for Gla-300 did not achieve statistical significance in the ‘with DDPIVi’ group, most likely due to smaller sample size and lower power than in the ‘without DPPIVi’ group. The lower number of participants in the ‘with DPPIVi’ subgroup, and hence the lower number of confirmed (≤3.9 mmol/L [≤70 mg/dL]) or severe hypoglycaemic events (Gla-300: 446 events for ‘with DPPIVi’ vs 3523 events for ‘without DPPIVi’; Gla-100: 807 events for ‘with DPPIVi’ vs 4390 for ‘without DPPIVi’), made it more difficult to observe significant differences between treatment groups.

Results from this analysis indicate that the concomitant use of DPPIVi therapy had a generally neutral effect on body weight change, with a small increase in body weight (0.3–0.8 kg) observed in all subgroups over 6 months of treatment. This is consistent with observations from previous studies of the co-administration of a DPPIVi and insulin [8–10]. The adverse event profile was generally similar between treatment groups and DPPIVi subgroups.

The limitations of this analysis are inherent to the post hoc nature. It should also be noted that the EDITION studies were not designed to address the question posed by this analysis; however, they were designed from the outset to allow data to be pooled for post hoc analyses. There were minor differences between the inclusion criteria in terms of HbA_1c_ between EDITION 2 (≥53 to ≤86 mmol/mol [≥7.0 to ≤10.0%]) and EDITION 3 (≥53 to ≤97 mmol/mol [≥7.0 to ≤11.0]). To address this, the model used to analyse HbA_1c_ change was adjusted on the baseline HbA_1c_ value. Moreover, participants from both studies had the same background antihyperglycaemic treatments. As mentioned above, the differences in the number of people in the DPPIVi subgroups may have limited data interpretation. Nevertheless, observations from this analysis may help guide combination treatment in people with type 2 diabetes who are on DPPIVi plus basal insulin therapy.

In conclusion, the comparable glycaemic control with lower hypoglycaemia risk with Gla-300 versus Gla-100 was observed irrespective of concomitant DPPIVi use in this population of people with type 2 diabetes. People with uncontrolled type 2 diabetes receiving a DPPIVi and basal insulin may benefit from Gla-300 therapy.

## Supporting information

S1 Table Daily basal insulin dose (U/kg) and change from baseline by visit and DPPIVi subgroup over 6 months of treatment (pooled mITT population). (DOC)

S2 Table Confirmed or severe hypoglycaemia over 6 months, by DPPIVi use (pooled safety population). (DOC)

S3 Table Adverse events over 6 months of treatment, by DPPIVi use (pooled safety population). (DOC)
