# Supplementary material for: The effect of concomitant DPPIVi use on glycaemic control and hypoglycaemia with insulin glargine 300 U/mL (Gla-300) versus insulin glargine 100 U/mL (Gla-100) in people with type 2 diabetes: A patient-level meta-analysis of EDITION 2 and 3
Source: PLoS One. 2018 Jan 25;13(1):e0190579. doi: 10.1371/journal.pone.0190579 (PMC5784896; doi:10.1371/journal.pone.0190579)
Supplement: S1 Table — (DOC) [file pone.0190579.s001.doc]

**S1 Table**. Daily basal insulin dose (U/kg) and change from baseline by visit and DPPIVi subgroup over 6 months of treatment (pooled mITT population)

|  | **Without concomitant DPPIVi use** | | **With concomitant DPPIVi use** | |
| --- | --- | --- | --- | --- |
|  | **Gla-300 (N=728)** | **Gla-100 (N=702)** | **Gla-300 (N=107)** | **Gla-100 (N=133)** |
| Baseline | 0.42 ± 0.27 | 0.43 ± 0.29 | 0.35 ± 0.28 | 0.38 ± 0.27 |
| Week 2 | 0.46 ± 0.27 | 0.47 ± 0.28 | 0.39 ± 0.28 | 0.40 ± 0.26 |
| Change from baseline to week 2 | 0.04 ± 0.04 | 0.04 ± 0.04 | 0.05 ± 0.03 | 0.03 ± 0.03 |
| Week 4 | 0.53 ± 0.27 | 0.53 ± 0.28 | 0.47 ± 0.29 | 0.46 ± 0.25 |
| Change from baseline to week 4 | 0.12 ± 0.08 | 0.10 ± 0.08 | 0.13 ± 0.08 | 0.08 ± 0.08 |
| Week 8 | 0.64 ± 0.29 | 0.62 ± 0.27 | 0.60 ± 0.31 | 0.54 ± 0.26 |
| Change from baseline to week 8 | 0.23 ± 0.15 | 0.18 ± 0.15 | 0.26 ± 0.16 | 0.17 ± 0.14 |
| Week 12 | 0.72 ± 0.31 | 0.67 ± 0.29 | 0.67 ± 0.31 | 0.59 ± 0.28 |
| Change from baseline to week 12 | 0.30 ± 0.21 | 0.24 ± 0.19 | 0.35 ± 0.22 | 0.22 ± 0.19 |
| Month 4 | 0.75 ± 0.33 | 0.69 ± 0.29 | 0.71 ± 0.34 | 0.59 ± 0.29 |
| Change from baseline to month 4 | 0.33 ± 0.23 | 0.25 ± 0.21 | 0.39 ± 0.26 | 0.22 ± 0.19 |
| Month 6 | 0.78 ± 0.34 | 0.71 ± 0.30 | 0.73 ± 0.35 | 0.59 ± 0.29 |
| Change from baseline to month 6 | 0.36 ± 0.26 | 0.27 ± 0.24 | 0.41 ± 0.28 | 0.23 ± 0.20 |

Data are pooled from EDITION 2 and EDITION 3, and are presented as mean ± SD. DPPIVi, dipeptidyl peptidase IV inhibitor; mITT, modified intention-to-treat; SD, standard deviation
