# Supplementary material for: The effect of concomitant DPPIVi use on glycaemic control and hypoglycaemia with insulin glargine 300 U/mL (Gla-300) versus insulin glargine 100 U/mL (Gla-100) in people with type 2 diabetes: A patient-level meta-analysis of EDITION 2 and 3
Source: PLoS One. 2018 Jan 25;13(1):e0190579. doi: 10.1371/journal.pone.0190579 (PMC5784896; doi:10.1371/journal.pone.0190579)
Supplement: S2 Table — (DOC) [file pone.0190579.s002.doc]

**S2 Table**. Confirmed or severe hypoglycaemia over 6 months, by DPPIVi use (pooled safety population)

|  | | | **Nocturnal (00:00–05:59 h)** | | | | | **Any time of day (24 h)** | | | | |
| --- | --- | --- | --- | --- | --- | --- | --- | --- | --- | --- | --- | --- |
|  |  |  | **Gla-300** | **Gla-100** | **RR** | **95% CI** | **Treatment-by-subgroup interaction p-value** | **Gla-300** | **Gla-100** | **RR** | **95% CI** | **Treatment-by-subgroup interaction p-value** |
| Confirmed (≤3.9 mmol/L [≤70 mg/dL]) or severe | % participants with ≥1 event | Overall* | 22.9 | 31.4 | 0.73 | 0.62 to 0.85 | 0.476 | 57.6 | 64.5 | 0.89 | 0.83 to 0.96 | 0.950 |
|  |  | Without DPPIVi^†^ | 22.6 | 31.4 | 0.71 | 0.60 to 0.85 |  | 57.3 | 63.7 | 0.90 | 0.83 to 0.97 |  |
|  |  | With DPPIVi^‡^ | 25.2 | 31.6 | 0.86 | 0.57 to 1.28 |  | 59.8 | 68.4 | 0.91 | 0.75 to 1.10 |  |
|  | Events per participant-year | Overall* | 1.6 | 2.5 | 0.64 | 0.48 to 0.85 | 0.276 | 10.1 | 13.3 | 0.77 | 0.65 to 0.91 | 0.978 |
|  |  | Without DPPIVi^†^ | 1.6 | 2.6 | 0.60 | 0.44 to 0.82 |  | 10.3 | 13.4 | 0.77 | 0.64 to 0.92 |  |
|  |  | With DPPIVi^‡^ | 1.8 | 2.1 | 0.95 | 0.45 to 2.03 |  | 8.8 | 12.5 | 0.77 | 0.49 to 1.23 |  |
| Confirmed (<3.0 mmol/L [<54 mg/dL]) or severe | %  participants with ≥1 event | Overall* | 6.8 | 9.8 | 0.69 | 0.50 to 0.95 | 0.710 | 18.3 | 25.4 | 0.72 | 0.60 to 0.86 | 0.218 |
|  |  | Without DPPIVi^†^ | 7.0 | 10.3 | 0.67 | 0.48 to 0.94 |  | 18.3 | 26.3 | 0.68 | 0.57 to 0.84 |  |
|  |  | With DPPIVi^‡^ | 5.6 | 7.5 | 0.77 | 0.29 to 2.06 |  | 17.8 | 20.3 | 0.96 | 0.56 to 1.64 |  |
|  | Events per participant-year | Overall* | 0.3 | 0.4 | 0.58 | 0.39 to 0.88 | 0.906 | 1.0 | 1.3 | 0.76 | 0.57 to 1.02 | 0.949 |
|  |  | Without DPPIVi^†^ | 0.3 | 0.5 | 0.57 | 0.37 to 0.88 |  | 1.1 | 1.4 | 0.75 | 0.56 to 1.00 |  |
|  |  | With DPPIVi^‡^ | 0.2 | 0.3 | 0.62 | 0.16 to 2.43 |  | 0.5 | 0.8 | 0.78 | 0.28 to 2.15 |  |

Data are pooled from EDITION 2 and EDITION 3. *Gla-300: N=838 (392.31 participant-years), Gla-100: N=844 (391.53 participant-years); ^†^Gla-300: N=731 (341.76 participant-years); Gla-100: N=711 (327.06 participant-years); ^‡^Gla-300: N=107 (50.55 participant-years); Gla-100: N=133 (64.48 participant-years). CI, confidence interval; DPPIVi, dipeptidyl peptidase IV inhibitor; RR, relative risk for % participants with ≥1 event or rate ratio for events per participant-year
