# Supplementary material for: The effect of concomitant DPPIVi use on glycaemic control and hypoglycaemia with insulin glargine 300 U/mL (Gla-300) versus insulin glargine 100 U/mL (Gla-100) in people with type 2 diabetes: A patient-level meta-analysis of EDITION 2 and 3
Source: PLoS One. 2018 Jan 25;13(1):e0190579. doi: 10.1371/journal.pone.0190579 (PMC5784896; doi:10.1371/journal.pone.0190579)
Supplement: S3 Table — (DOC) [file pone.0190579.s003.doc]

**S3 Table. Adverse events over 6 months of treatment, by DPPIVi use (pooled safety population)**

|  | **Without concomitant DPPIVi use** | | **With concomitant DPPIVi use** | |
| --- | --- | --- | --- | --- |
| **n (%)** | **Gla-300 (N=731)** | **Gla-100 (N=711)** | **Gla-300 (N=107)** | **Gla-100 (N=133)** |
| Participants with any TEAE | 413 (56.5) | 367 (51.6) | 71 (66.4) | 84 (63.2) |
| Participants with any treatment-emergent SAE | 32 (4.4) | 30 (4.2) | 7 (6.5) | 11 (8.3) |
| Participants with any TEAE leading to death | 2 (0.3) | 1 (0.1) | 1 (0.9) | 0 (0) |
| Participants with TEAE leading to permanent treatment discontinuation | 10 (1.4) | 7 (1.0) | 1 (0.9) | 2 (1.5) |

Data are pooled from EDITION 2 and EDITION 3. DPPIVi, dipeptidyl peptidase IV inhibitor; TEAE, treatment-emergent adverse event; SAE, serious adverse event
